# Central venous pressure as a method of optimising atrio-ventricular delay after cardiac surgery

**DOI:** 10.1371/journal.pone.0310905

**Published:** 2025-01-17

**Authors:** Alexander Tindale, Ioana Cretu, Naomi Gomez, Ross Haynes, Hongying Meng, Mark J. Mason, Darrel P. Francis

**Affiliations:** 1 Department of Cardiology, Harefield Hospital, Guys & St Thomas’ Foundation Trust, London, United Kingdom; 2 National Heart and Lung Institute, Imperial College London, London, United Kingdom; 3 Brunel University London, Uxbridge, United Kingdom; UN Mehta Institute of Cardiology and Research Center, INDIA

## Abstract

**Introduction:**

Haemodynamic atrioventricular delay (AVD) optimisation has primarily focussed on signals that are not easy to acquire from a pacing system itself, such as invasive left ventricular catheterisation or arterial blood pressure (ABP). In this study, standard clinical central venous pressure (CVP) signals are tested as a potential alternative.

**Methods:**

Sixteen patients with a temporary pacemaker after cardiac surgery were studied. AV delay optimisation was performed by alternating between a reference AVD of 120ms and tested settings ranging from 40 to 280ms, with 8 replicates for each setting. Alongside (a) the raw data, three methods of correcting for respiration were tested: (b) limiting analysis to a respiratory cycle, (c) asymmetric least squares (ALS) and (d) discrete wavelet transform (DWT). The utility of a quality control step was tested.

**Results:**

CVP signals were a mirror image of the systolic ABP signals: The four R values were -0.674, -0.692, -0.631, -0.671 respectively (all p<0.001). With quality control, the mirror image was best for DWT (R = -0.76, p<0.001), with the CVP and ABP optima agreeing well (R = 0.78, p<0.001). The automated quality control signal correctly predicted the gap between the AVD optima calculated from ABP and CVP (R = 0.8, p<0.001).

**Conclusions:**

Central venous pressure signals could be used to optimise AVD, because they have a reliable inverse relationship with ABP when pacemaker settings undergo protocolised testing. However, protocols need careful design to circumvent spontaneous biological variability.

## Introduction

In a patient shortly after cardiac surgery, cardiac function is often precarious. Many such patients have temporary dual-chamber pacing, which can have beneficial haemodynamic effects [1]. We know that in stable outpatients with chronic heart failure, AV delay adjustment delivers large haemodynamic effects, for example dwarfing any impact of VV delay adjustment in cardiac resynchronisation therapy (CRT) [2]. Short AV delay interferes with the atrial component of ventricular filling, and excessive AV delay truncates passive ventricular filling [3].

The AV delay can be optimised using measures in the arterial circulation, such as arterial blood pressure [4]. However, arterial blood pressure sensing is not currently available in implantable form. CVP sensing is much more realistic since the pacemaker leads pass through the central veins.

CVP is monitored routinely in patients recovering from cardiac surgery. An isolated increase in cardiac function may be expected to reduce CVP at the same time as it increases systemic blood pressure [5]. Whether these effects are sufficiently similar for CVP to be used for AVD optimisation is unknown.

CVP varies more than arterial blood pressure during the respiratory cycle, however, both in absolute and proportional terms. Any signal from changes in CVP, therefore, may be lost in the noise of the respiratory cycle. For this reason, it is conventional to standardise CVP measurement as performed at end expiration [6]. Restricting measurements to this narrow window of time, however, greatly reduces the amount of data available for an optimisation process which may require many hundreds of measurements to give a sufficiently precise answer.

An algorithm that can compensate for respiration could, therefore, enhance the efficiency of using CVP for AVD optimisation.

This study tests whether CVP behaves in a reproducibly inverse pattern to arterial blood pressure to be used in AVD optimisation, and the utility of various respiratory noise compensation methods to improve the efficiency of the optimisation process.

## Methods

### Patient demographics

Sixteen inpatients with temporary pacing systems were studied within 72 hours of cardiac surgery whilst on the Intensive Care Unit: 14 with underlying sinus rhythm and 2 who were pacing dependent. All patients had epicardial leads attached to the right atrium and right ventricle. The patients ranged from 41 to 80 years in age (mean 71 years). Twelve were male, four were female. The left ventricular function ranged from 31% to 65%, median 55%. Nine patients underwent coronary artery bypass surgery alone, three aortic valve and root replacement, two isolated aortic valve replacements, one coronary artery bypass surgery and mitral valve repair and one isolated tricuspid valve replacement. Patient-level data is available in the supplementary materials available from the online GitHub repository.

### Recording haemodynamic signals

Invasive blood pressure was measured using Edwards Lifesciences TruWave pressure transducers connected to a Boston Scientific BARD amplifier. ECG signals were also recorded by the Boston Scientific system. Analog signals were taken by a National Instruments DAQ card (National Instruments, TX, USA) and then acquired digitally using Labview software (also NI). The central venous catheters were inserted through the internal jugular vein with the tip sited in the superior vena cava. All invasive arterial pressure readings were taken from the radial artery.

### Protocol for measuring relative pressure changes during AV delay adjustment

Subjects were atrially paced at either 90 beats per minute or 10 beats per minute above sinus rhythm, whichever was higher. These relatively high heart rates were chosen both pragmatically (as the patients were within 72 hours of cardiac surgery) and because previous work has shown that changes in AV delay are more evident at higher heart rates [4].

The AV delays tested were 40ms, 80ms, 120ms, 160ms, 200ms, 240ms, and 280ms. Each delay was tested against a reference state, which was 120ms. Eight transitions between resting and tested state were conducted for each AVD. Testing was terminated for each subject at the first point after intrinsic conduction occurred or after a tested AVD of 280ms. For example, if a subject had intrinsic AV nodal conduction at a tested AV delay of 240ms then this was used as the final tested AV delay.

### Calculation of the effects of each AV delay

For each transition, the change in blood pressure for a window immediately before the transition and a window immediately after the transition. In the standard method, the window length was 6 beats. One of the proposed respiratory corrections was to vary the window length on an individual patient basis, to match one respiratory cycle of that patient.

Eight replicate transitions were conducted for each tested AVD (e.g. reference to 160ms), and calculated the mean and standard error of the mean (SEM) for each AVD.

### Correcting for respiration

**Fig 1:**
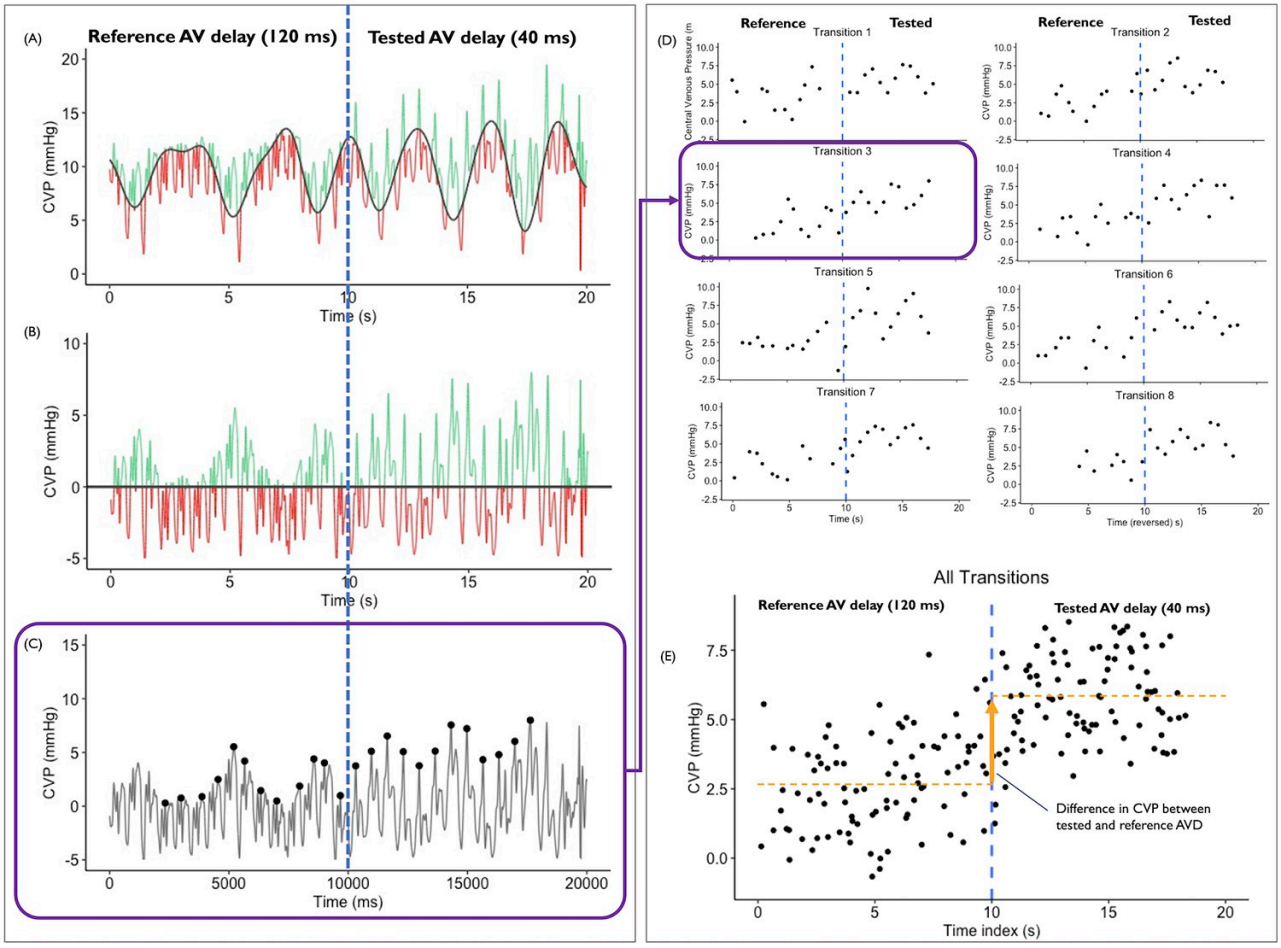
Steps in obtaining peak CVP data around transition points. (A) Original CVP signal around a pacing transition from DDD with an AVD of 120ms to 40ms. The overlying signal (solid black line) is the respiratory variation as calculated by DWT. (B) The Corrected CVP where the respiratory variation has been subtracted from the original signal. (C) Peak identification of 2 peaks per cardiac cycle. (D) For each replicate (labelled transition 1–8), the black dots represent the peak for 6 heartbeats each side of the transition. (E) Amalgamation of all the data points from (D) showing the difference between tested and reference AV delays.

The second and third methods of correction for respiration were Discrete Wavelet Transform (DWT) and Asymmetric Least Squares (ALS). DWT was performed in *R* using the "Waveletcomp" package [7]. An example of a section of an uncorrected CVP trace is shown in Fig 1. The respiratory cycle has been fitted (shown by the red line). The reconstructed respiratory trace is then subtracted from the uncorrected CVP trace to form the DWT-corrected CVP trace in Fig 1. A similar process was conducted for ALS [8].

### Calculating the signal-to-noise ratio

**Fig 2:**
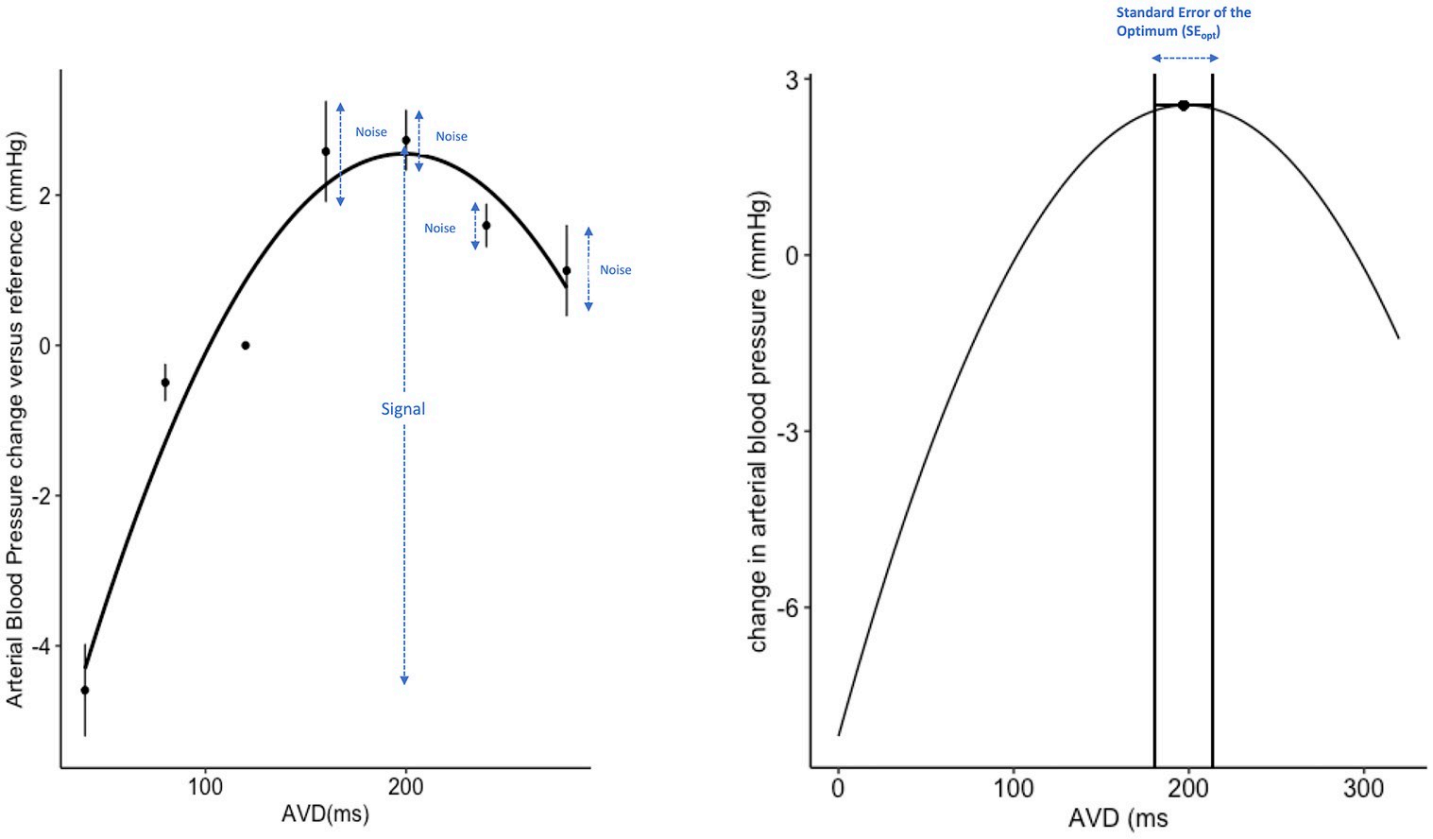
Different methods of measuring uncertainty. (Left panel): Calculating the Signal-to-noise (STN) ratio. The STN was calculated for each patient by dividing the maximum difference between best and worse settings (“The signal”) by the average of the SEMs around each point (“the noise”). The STNs for each were then averaged to calculate the overall STN for each method. (Right panel): Demonstration of the Standard Error of the Optimum.

The signal-to-noise ratio was calculated in line with previously published work [9]. The maximum between the best and worst tested AV delays (the signal) was divided by the mean SEM for the transitions at that particular AV delay (the noise). This is shown graphically in Fig 2.

### Quality control protocol

In line with methodology previously published [10], a secondary analysis was performed using only data which passed two quality control tests.

The initial step is to calculate the clinical optimum from the quadratic equation governing the parabola. Assuming that:

$$y={ax}^{2}+bx+c$$

Then the optimum occurs when the gradient is zero, and hence differentiating y with respect to x:

$$\frac{dy}{dx}=2ax+b=0$$

And therefore:

$$x_{opt}=\frac{-b}{2a}$$

The next step is to ensure that the parabolas are oriented correctly (i.e. for arterial blood pressure, the coefficient for the AVD^2^ should be negative, and positive for the fitted CVP parabola). The final test is that the standard error of the optimum (SE_opt_) should be less than 50ms on average for that patient (Fig 2). This is twice as stringent as previous studies [10]. The SE_opt_ was calculated in line with previous published methodology [11]. The rationale behind this is to mitigate for excessive noise in the data being analysed.

### Statistical analysis

Data was processed using custom automated software written by the authors in both R version 3.6.2 (R Project, Vienna, Austria) and Python (Python Foundation, Wilmington, Delaware, USA). All statistical analysis was performed using *R*.

### Ethics

The study was compliant both with good clinical practice guidelines and the Declaration of Helsinki. The data was collected as part of the PACESIM trial (ISRCTN15383573) with ethical approval given by South West—Cornwall & Plymouth Research Ethics Committee. All participants signed written, informed consent. All participants were consented and studies between 1^st^ July 2021 and 22^nd^ March 2023.

## Results

### (1) Effect of changing the AV delay on ABP

The optimum tested AVD as defined by fitting a parabola to the ABP values varied from 100 to 320 ms. For all patients, the median optimal AV delay was 195 (IQR 166–223) ms and from those who passed quality control 198 (173–222) ms. In total, 11/16 patients passed both quality control metrics.

Across all patients, the mean BP difference between the best tested AVD and the reference AVD was 4.3 +/- 0.8 mmHg and the mean difference between best and worst tested AVD was 11.4+/-1.1 mmHg. For patients passing quality control, these were 3.6 +/- 1.0 and 11.7 +/- 1.3 mmHg respectively.

44/85 (52%) of setting changes away from a reference of 120ms resulted in a negative effect on arterial blood pressure.

### (2) Quality control using standard error of the optimum (SE_opt_)

Of the 64 patient-level analyses using different correction methods, 43/64 (67%) passed both quality control measures. 11 failed for incorrectly oriented curves and a further 10 for SE_opt_ being >100ms (3 for CVP, 8 for ABP). For those passing quality control, the median SE_opt_ was 16ms (9–34) for ABP and 17ms (8–37) for CVP.

**Fig 3:**
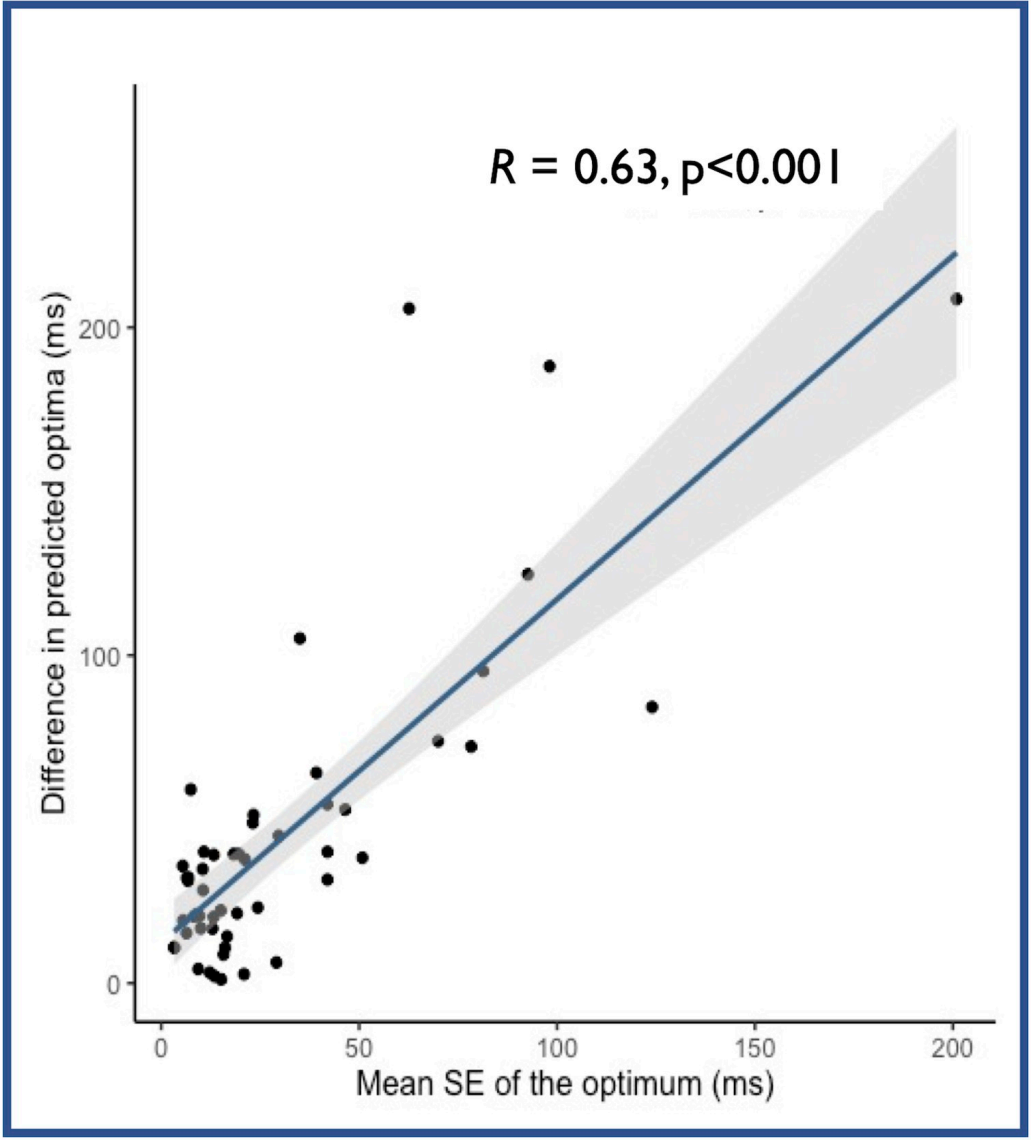
The relationship between SEMopt and the difference in the predicted optima using ABP and CVP with all correction methods. As the uncertainty of the optimal AV delay increases (higher SE_opt_), the divergence between the optima predicted by CVP and ABP increases. R value derived using Spearman’s method due to heteroskedasticity in data.

Calculation of the SE_opt_ was tested to establish its utility in predicting the reliability of the optimisation process: namely how different the results of the two optimisation methods would be. A strong relationship was found between the SE_opt_ and the size of the discrepancy between the ABP and CVP-based optima (R = 0.63, p<0.001, Fig 3). This means that a small SE_opt_ is a good indicator that the optimum is reliable.

### (3) Correlation between ABP and CVP measurements using different correction methods

**Fig 4:**
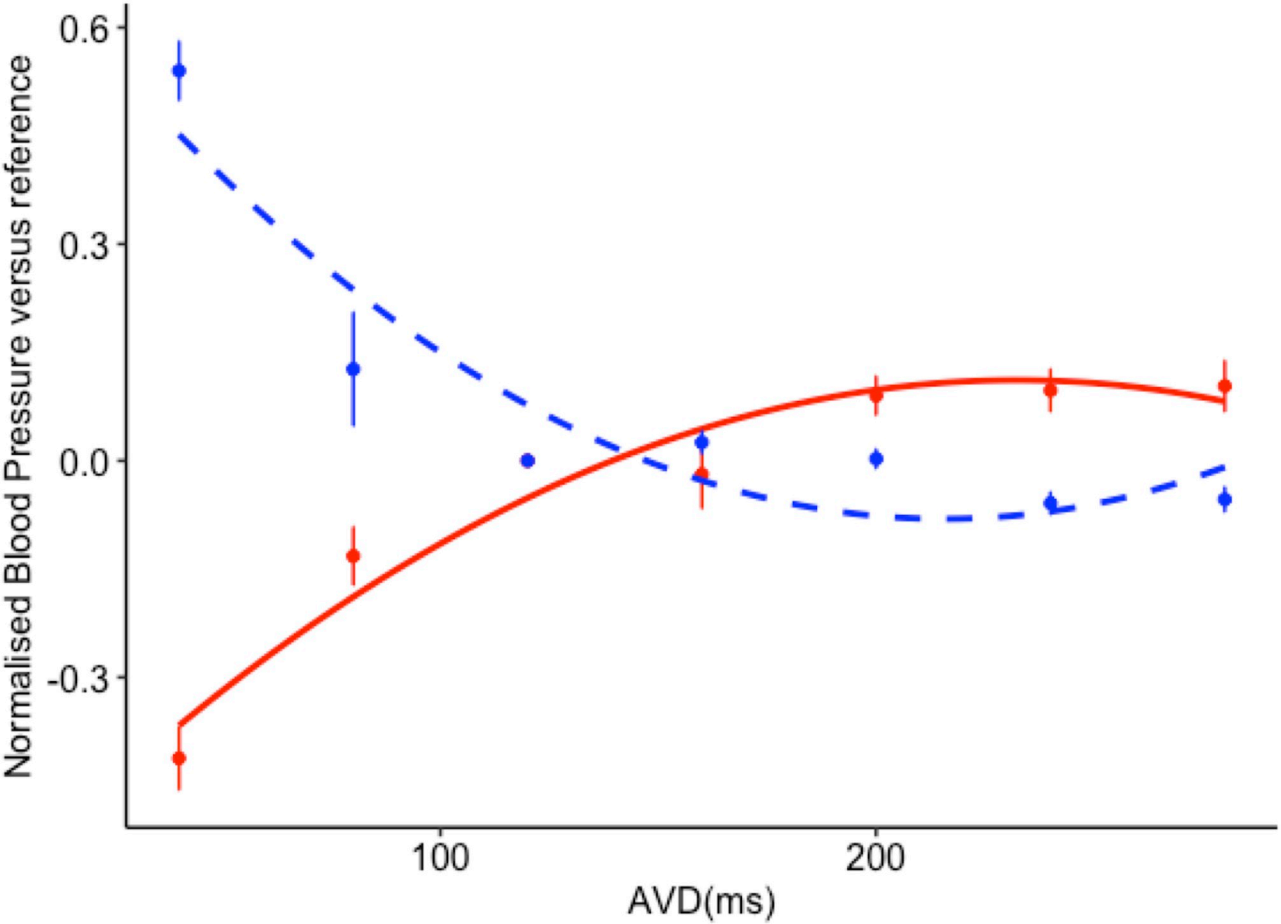
The effect of changing AV delay on normalised central venous pressure (-—-) and arterial blood pressure (solid line).

In general, the curve of ABP vs AVD was approximately an inverted copy of the curve of peak CVP versus AV delay, as shown in the example patient in Fig 4.

The correlation between CVP changes and ABP changes for each transition is shown in supplementary materials available from the online GitHub repository. The correlations were stronger when using peak CVP rather than mean CVP, and this was true for all methods of correction. The strength of the correlation ranged between an R value of 0.631 for ALS to 0.692 when using one respiratory cycle. All were statistically significant.

**Fig 5:**
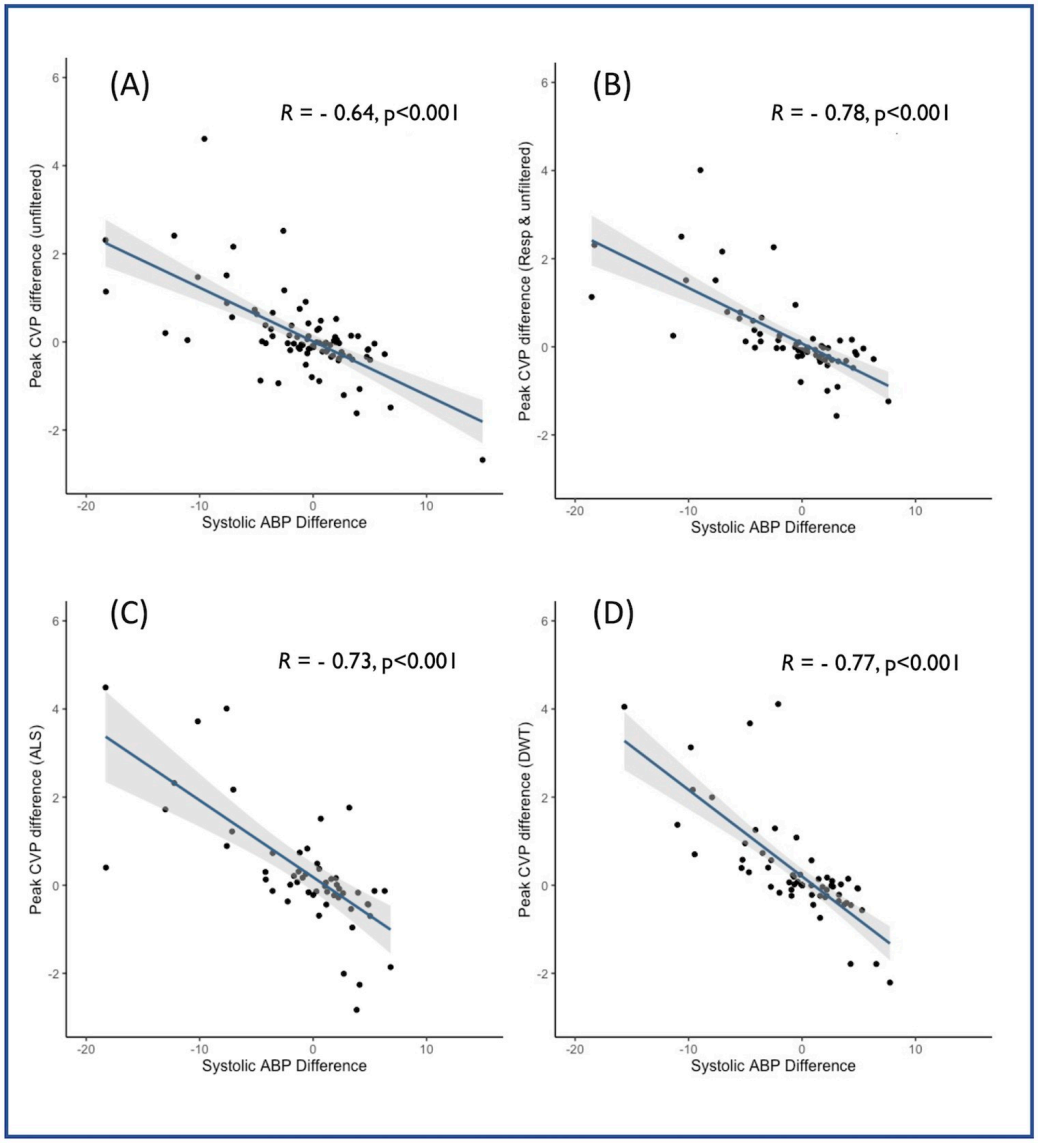
Relationship between Peak CVP and ABP using different methods of respiratory correction in patients passing quality control: (A) No respiratory correction(6 beats). (B) No correction but one respiratory cycle length around transition. (C) Asymmetric Least Squares (ALS, 6 beats). (D) Discrete Wavelet Transform (DWT, 6 beats). R value derived using Spearman’s method.

The relationship strengthened when examining only those patients who passed the quality control algorithm (Fig 5). The strongest relationship was between ABP and CVP as corrected by DWT (R = 0.76, p<0.001, Fig 5 panel D).

### (4) Signal-to-noise ratio of ABP and CVP measurements

**Fig 6:**
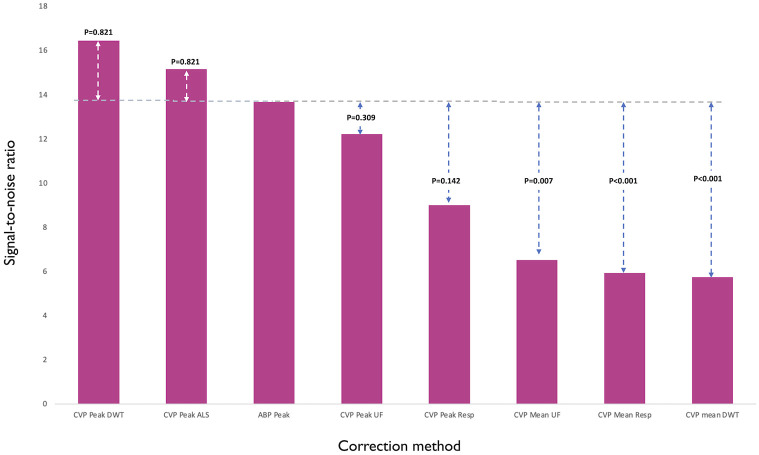
Median signal-to-noise ratio by each method of correction for different transition lengths. DWT = Discrete Wavelet Transform. NC = No Correction. ALS = Asymmetric Least Squares. Resp = Number of beats corresponding to one respiratory cycle. 6 = 6 beats around transition.

For peak CVP, regardless of the method of correction, the signal to noise ratio was similar to that of ABP. In contrast, for mean CVP, regardless of the method of correction, the signal-to-noise ratio was worse than that for ABP (Fig 6).

### (5) Correlation between the calculated optimal AV delay as calculated by CVP and ABP

**Fig 7:**
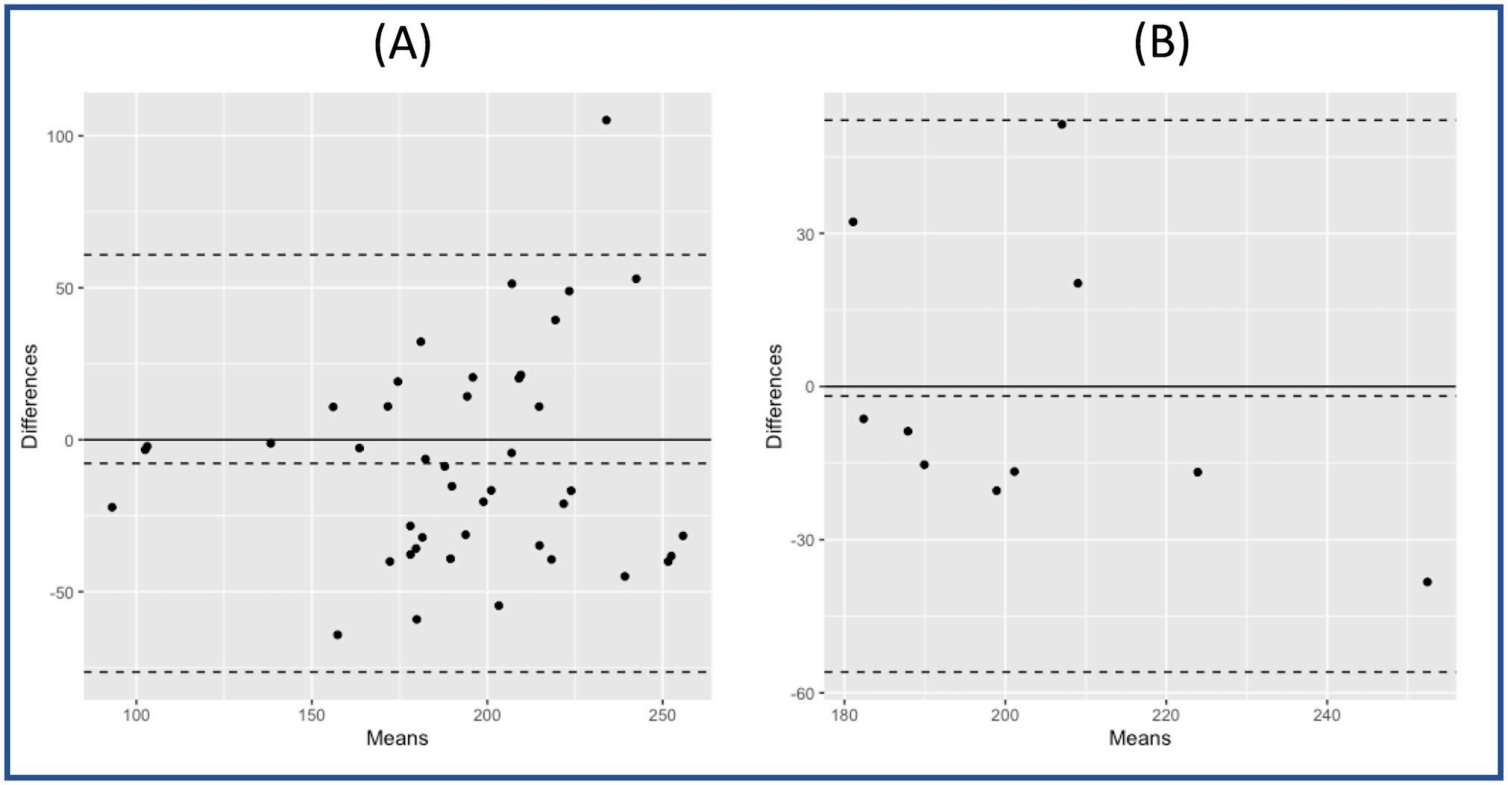
Bland Altman plots showing agreement between the optimal AVD as defined by both CVP and ABP: (A) for all correction methods, and (B) for CVP corrected by DWT.

Across all methods of CVP correction, there was a strong relationship between the optimum predicted by ABP and any CVP measurement (R = 0.64, p<0.001). The strongest relationship was in patients who had respiration corrected using DWT (R = 0.78, p = 0.005). Bland-Altman plots show a good agreement between the optimal AVD predicted by either ABP or CVP for each patient (Fig 7).

### (6) Absolute effect of optimised pacing on CVP

For patients who passed quality control, this difference between best and worst settings was 4.15 (0.87) mmHg (p<0.001). The best settings reduced the CVP by 0.86 (0.30) mmHg compared to the reference AV delay (p = 0.008)

### (7) Pressure changes for respiration

Using DWT to map the underlying respiratory cycle, the respiration change for CVP was 2.3mmHg (SD 1.6) and ABP was 2.5 (SD 1.6) mmHg.

## Discussion

This study has shown that when AV delay is manipulated, central venous pressure goes down when arterial blood pressure goes up. Automatic correction for respiration makes this relationship even stronger, and use of peaks rather than means further increased the signal-to-noise ratio. There are some patients, however, in whom the noise overwhelms the signal. Fortunately, these can be recognised automatically by a mathematical quality control step.

### Central venous pressure as a target for optimisation

Signals on the arterial side of the cardiovascular system are the most obvious targets for optimisation of pacemaker function because the respiratory fluctuations in the arterial circulation (e.g. blood pressure) tend to be a relatively small fraction of the overall value. The venous side of the cardiovascular system is easier to access, however, in patients receiving pacing from either permanent or temporary pacing systems.

This study suggests that CVP is a plausible alternative to ABP as an optimisation target. Surprisingly, the signal to noise ratio is not worse for CVP than ABP. This may be because, whilst the respiratory fluctuation is large in the context of the average value of the CVP, it is not necessarily long compared to the impact of the pacing. What matters for precise optimisation is that the noise is smaller than the difference between pacing settings, not how it compares to the average value.

The size of the respiratory fluctuation in CVP was only 2.3mmHg (SD 1.6), which was no different from the size of the respiratory fluctuation in ABP (2.5mmHg, SD 1.6). This initially surprised us, but in retrospect it is consistent with the mechanism of the fluctuation being identical in both, namely a transmitted change in intrathoracic pressure caused by movement of the diaphragm and other respiratory muscles.

### Automatically determining the uncertainty of the optimum

This study also shows that it is possible to estimate the AVD uncertainty of an optimisation (SE_opt_) using data within the optimisation itself. The concept has been described in principle previously [11], but this study verifies the applicability of the method in both arterial and venous circulations.

This is important because an optimisation with a wide uncertainty in optimal AV delay may be more harmful than beneficial [12]. It is therefore important for any system to be able to recognise automatically that the uncertainty in the AV delay optimum is wide and conduct more measurements. These measurements may take additional time and energy (a concern particularly for an implanted system), and therefore routinely making unnecessarily large numbers of measurements may also be unacceptable.

This automated judgement is based on two steps. In the first step, if the curve is inverted this certainly represents noise swamping the signal and therefore more data is required. In the second, a formula based on the vertical error (variation between replicate measurements at the same AVD) is used to estimate the horizontal error (uncertainty of the optimum AVD), as shown in Fig 2.

When the horizontal error estimated by this method is small, the estimated AVD optimum can be trusted to be correct, in that the same AVD optimises both ABP and CVP (Fig 3).

### Correcting for respiration

Respiration may be particularly disruptive for measurements in the post-operative intensive care setting. A common solution in clinical practice is to measure CVP at a standardised point in the respiratory cycle, most conveniently end-expiration [6]. However, if the protocol requires hundreds of measurements, this requirement (because it discards the great majority of the respiratory cycle) makes the protocol take many times longer.

Three methods for respiratory correction were tested: (i) measuring for exactly one respiratory cycle before the transition and one cycle after; (ii) subtracting a smoothed respiratory signal calculated by asymmetric least squares (ALS); (iii) the same subtraction principle using discrete wavelet transform (DWT).

DWT performed best, though in this study it was used in a different manner to its previous uses in cardiology. Rather than using DWT to extract the desired low-frequency component from noisy data such as ECG [13], DWT was used to extract the unwanted low-frequency component (respiration) so that it could be subtracted from the pressure signal.

DWT is more suited than Fourier transformation because it does not assume that the respiratory pattern and frequency is consistent throughout the recording [14].

The ALS has the theoretical advantage of not using any preconceptions of the smoothing shape [8]. In practice, however, its results were poorer than using a single-length respiratory cycle. In the noisy post-surgical data in this study, sudden and irregular inspirations caused large spikes in CVP that the ALS was not able to correct fully.

### Using SE_opt_ is a useful adjunct to examine the relationship between CVP and ABP

The signals in this study, SE_opt_ of 17ms (CVP) & 16ms (ABP), are no more noisy than previous studies with arterial signals: SE_opt_ 26ms for photoplethysmography and 53ms for laser doppler values [10]. Data in this study was taken from invasive haemodynamic measurements and hence is likely to have less noise and thus a higher signal-to-noise ratio. Both the SE_opt_ and signal to noise ratios allow a comparison between the information content of different optimisation methods.

### Limitations and avenues for further research

This pilot study is from a small cohort of patients in a single setting with temporary pacing after cardiac surgery. Therefore, the findings may not apply as readily to stable patients with permanent devices in the outpatient setting.

Furthermore, in this study beat-to-beat CVP measurements were compared only to arterial blood pressure measurements rather than echocardiographic parameters, which are often considered the standard for AV delay optimisation [15]. Therefore, we cannot state for certain how these CVP readings relate to echocardiographic findings during AV delay manipulation. However, with echocardiography the measurement of each beat is laborious, and it is unusual to measure a large enough number of beats to prevent the beat-to-beat variability from exceeding the difference between the settings. These findings could be validated with an echocardiography study using automated measurements of doppler velocity time interval which we could confidently rely on as an index of changes in flow [16].

Finally, this study is largely descriptive in nature, focussing on the correlation between central venous pressure and arterial blood pressure after correction. There is the possibility that findings where we have interpreted causal relationships may be driven instead by unknown cofounders, especially given the heteroskedasticity in some of the data. Future work could involve the use of different analytic models, including generalised least squares, to give further heft to the inferences that have been made.

## Conclusions and further clinical applications

Central venous pressure may be a plausible alternative to arterial blood pressure as a target for haemodynamic optimisation of AV delay in dual-chamber pacing. However, automated processing is essential. First, the effect of respiration must be minimised, either by using an exact multiple of a respiratory cycle or by applying a mathematical correction algorithm. Second, the protocol needs sufficient replications to have an acceptably narrow SE_opt_: where this is not the case, the resulting estimated optimum must not be relied upon.

At the most basic level, this knowledge could be used to optimise temporary pacemakers post-surgically when the invasive arterial blood pressure is not available. CVP could, therefore, be explored as an optimisation marker in implanted devices (including CRT) which have leads inside the central veins. Pressure sensors could directly measure central venous pressure, and appropriate signal processing could attenuate the effect of respiration and automatically ensure data reliability.

## Supporting information

S1 Checklist Human participants research checklist. (DOCX)
